# Cytopathological Features of Extensive Bilateral Pleural Effusions in Metastatic Prostate Cancer: Report of a Rare Case

**DOI:** 10.7759/cureus.58597

**Published:** 2024-04-19

**Authors:** Hehua Huang, Caroline Yap

**Affiliations:** 1 Pathology and Laboratory Medicine, Harbor-UCLA (University of California Los Angeles) Medical Center, Torrance, USA

**Keywords:** serous fluid, nkx3.1, prostatic acid phosphatase (pap), prostate-specific antigen (psa), cytopathology, malignant pleural effusions, metastatic prostate adenocarcinoma, prostate cancer

## Abstract

We report a rare case of a 59-year-old male with a history of metastatic prostate cancer presenting with acute onset dyspnea due to extensive bilateral pleural effusions. This case highlights the rarity of metastatic prostate cancer with pleural involvement and underscores the importance of accurate diagnosis using cytopathology and immunohistochemical staining.

## Introduction

Prostate cancer is a common malignancy among men, with the bone being the most frequent site of metastasis [1]. Pleural involvement in metastatic prostate cancer is an extremely rare event, with an estimated prevalence of less than 1% [2,3]. This is an unusual case of metastatic prostate adenocarcinoma presenting as extensive bilateral pleural effusions. We discussed the cytopathologic features and immunohistochemical staining results, which confirmed its origin from the prostate.

## Case presentation

A 59-year-old male with a known history of metastatic prostate cancer presented to the emergency department experiencing acute dyspnea. The patient was previously diagnosed with prostate adenocarcinoma with bone metastasis at an external hospital; however, detailed histopathological information of the primary tumor and the Gleason score were not available. He underwent chemotherapy two years prior, received androgen deprivation therapy (leuprolide) and radiation treatment, and was recently deemed eligible for a new chemotherapy regimen due to pancytopenia. Approximately 10 days before this presentation, he was discharged from another hospital following a left thoracentesis that extracted 1.5 liters of pleural fluid.

On physical examination, the patient was tachycardic and tachypneic with decreased breath sounds and was noted to have abdominal distention and pitting edema of the lower extremities. Chest X-ray and CT scan showed large bilateral pleural effusions with overlying atelectasis and right-sided patchy opacities. CT scan of the abdomen and pelvis revealed ascites, anasarca, scattered hypodensities throughout the liver likely representing metastatic disease, and severe diffuse bony metastatic disease.

Diagnostic and therapeutic right thoracentesis yielded 700 ml pleural fluid and revealed malignant cells. Immunostaining results confirmed the diagnosis of metastatic adenocarcinoma originating from the prostate.

Cytopathology

**Figure 1:**
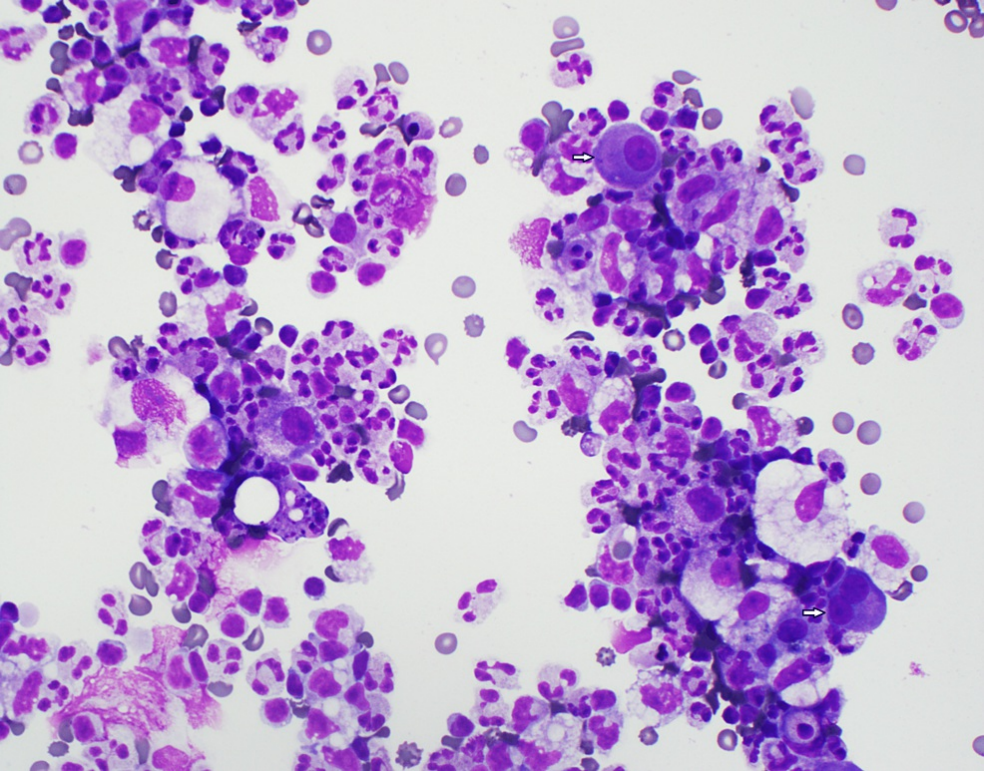
Cytospin of pleural fluid prepared with Diff-Quik stain Against a backdrop of neutrophils, histiocytes, and erythrocytes, there is a notably large cell with a distinct nucleus and pronounced nucleoli (top arrow), accompanied by a large binucleated atypical cell also with conspicuous nucleoli (bottom arrow).

The pleural fluid was subjected to a meticulous cytopathological examination. The direct smear (Figure 1) displayed large, atypical cells interspersed among neutrophils, erythrocytes, and reactive histiocytes. These atypical cells stood out due to their relatively large size (approximately 2-3 times the size of red cells), eccentric nuclei, smooth nuclear contours and prominent nucleoli. A distinct perinuclear halo was also evident around these cells. Intriguingly, some cells exhibited overlapping nuclear borders and were binucleated (as seen in Figure 1, bottom arrow cell).

**Figure 2:**
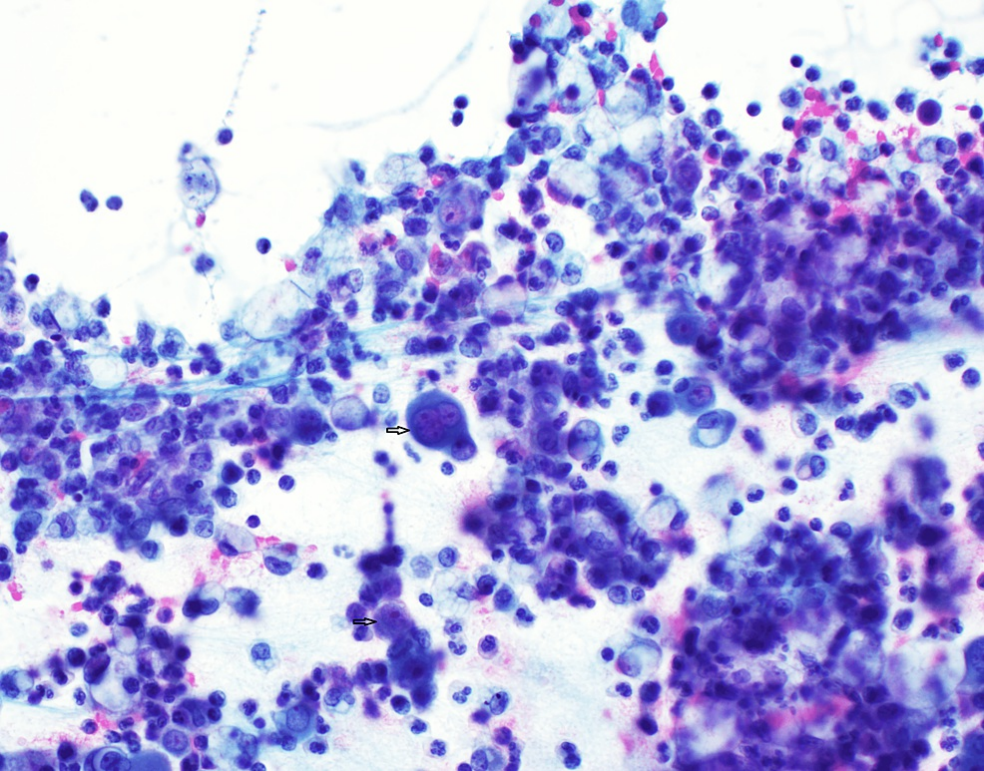
Direct smear of pleural fluid In the background of inflammatory cells, one cell stands out with three to four overlapping nuclei. This multinucleated cell has prominent nucleoli and high nucleus to cytoplasmic (N/C) ratio.

In the Cytospin preparation (Figure 2), set against an inflammatory cell background, certain cells were discernible due to their three to four overlapping nuclei. These cells were marked by large nuclei, conspicuous nucleoli, and a high nuclear-to-cytoplasmic (N/C) ratio.

**Figure 3:**
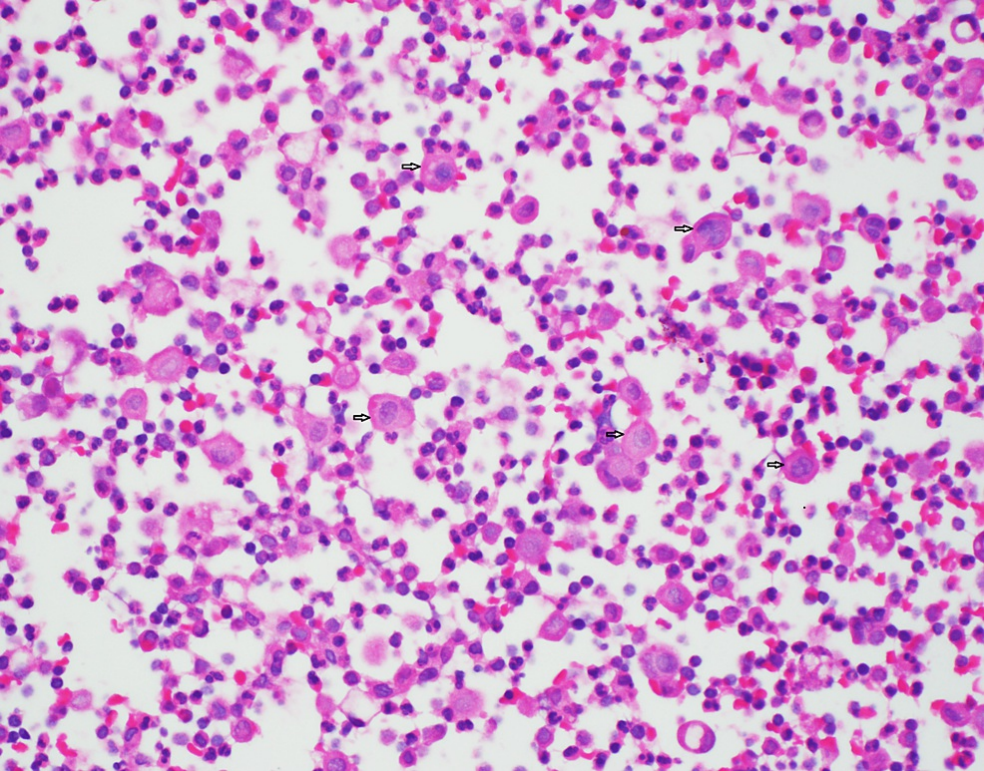
H&E stained cell block of pleural fluid Dispersed cells demonstrate moderate nuclear atypia. H&E: Hematoxylin and Eosin

A Hematoxylin and Eosin (H&E) stained cell block (Figure 3) further spotlighted dispersed cells with moderate atypia, with some being binucleated. While their nuclei were prominently larger (around two to three times the size of lymphocytes), their surrounding cytoplasm varied from loose to vacuolated. Notably, these cells suggested a metastatic carcinoma but lacked the cohesive gland formation often associated with adenocarcinoma.

**Figure 4:**
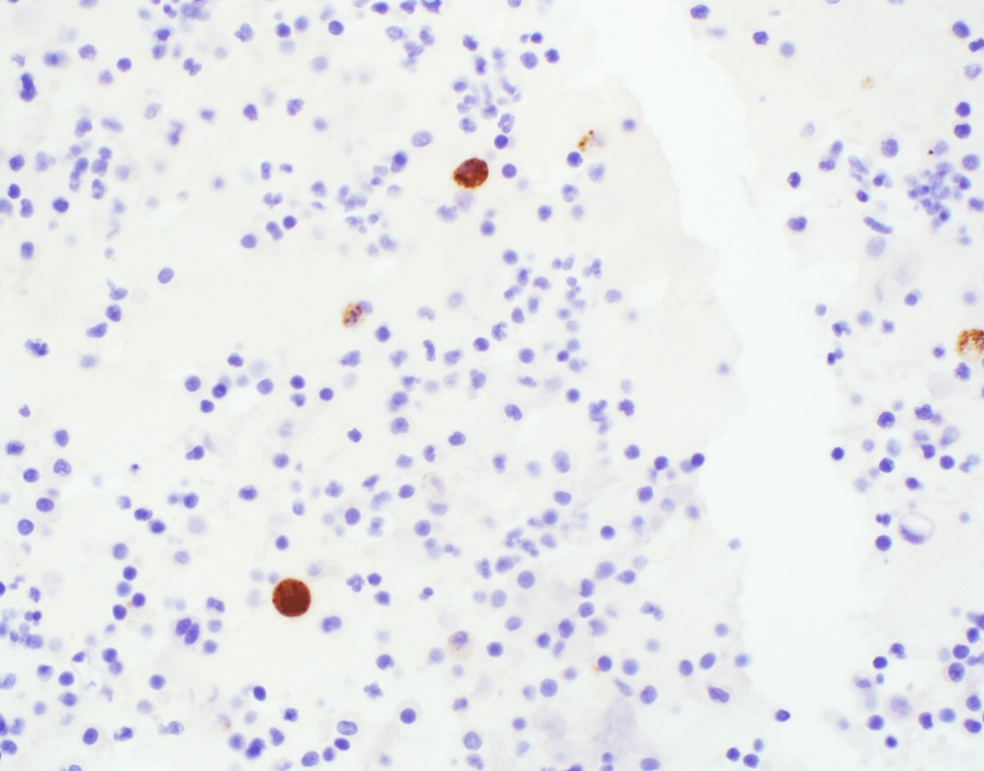
Immunohistochemical staining for PAP Positive result. PAP: Prostatic Acid Phosphatase

**Figure 5:**
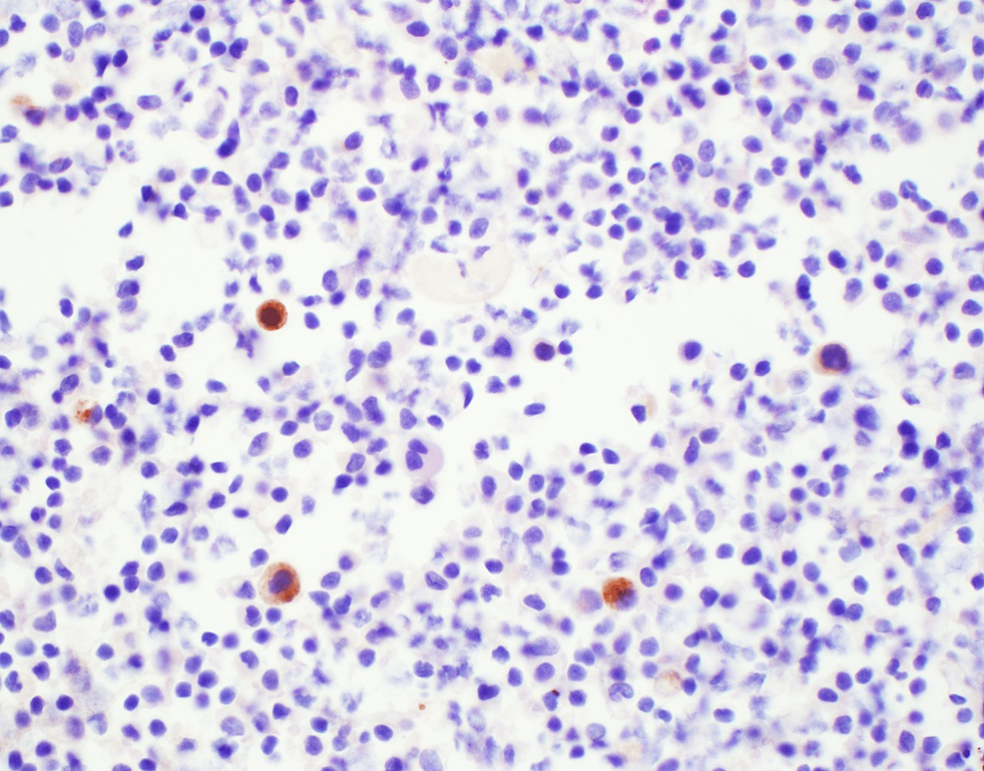
Immunohistochemical staining for PSA Positive result. PSA: Prostate-Specific Antigen

**Figure 6:**
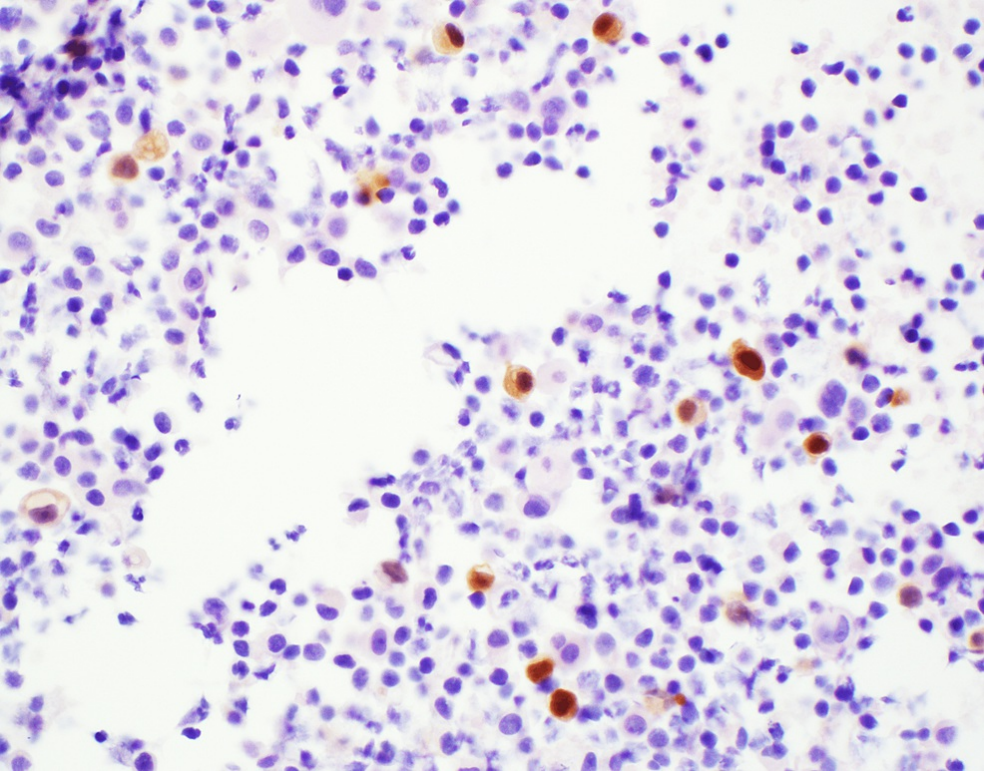
Immunohistochemical staining for NKX3.1 Positive result.

Immunohistochemical staining was instrumental in guiding our diagnostic insights, emphasizing the prostate cancer lineage. Positive staining for both Prostatic Acid Phosphatase (PAP) and Prostate-Specific Antigen (PSA) was observed (Figures 4-5). Additionally, the presence of the NKX3.1 marker, which is highly specific to prostate tissue, was confirmed (Figure 6). Together with the positive results for MOC-31 and other markers, these insights indisputably confirmed a diagnosis of metastatic prostate adenocarcinoma.

Differential diagnosis of metastatic prostate cancer in serous fluids can be intricate, given the potential overlap of cytopathological features with other malignancies or even reactive cells. The non-cohesive pattern we observed, characterized by large, vacuolated cytoplasm and mild to moderate nuclear atypia, can sometimes mirror that of reactive mesothelial cells in a pleural effusion. Markers such as calretinin, WT-1, and podoplanin (D2-40) can be employed to rule out mesothelial cells. In this case, the negative staining with calretinin ascertained that the atypical cells were not mesothelial in nature. Additionally, the marker CD68 aided in distinguishing macrophages, reinforcing that the cells under our lens were truly malignant. The absence of CK7 and CK20 staining further diminished the probability of gastrointestinal or lung adenocarcinoma origin. The epithelial malignancy was confirmed with MOC-31 staining. The combined insights from the patient's history, distinctive cytopathological features, and the positive staining for PAP/PSAP, PSA, and NKX3.1 unequivocally pinpointed the diagnosis of metastatic prostate adenocarcinoma. Though other immunohistochemical markers, such as Napsin A (to exclude a lung primary) are available, they weren't deemed necessary given the patient's history of prostate adenocarcinoma.

To conclude, this case underscores the value of a meticulous approach in cytopathology. Through the synergy of cytopathological and immunohistochemical analyses, a final diagnosis of metastatic prostate adenocarcinoma was attained.

Management and outcome

The patient's dyspnea and pleural effusions were managed with thoracentesis and indwelling pleural catheter placement. He was also started on palliative chemotherapy for his metastatic disease. However, the patient's condition continued to decline, and he ultimately succumbed to his illness only four days after admission and pleural fluid collection.

## Discussion

Prostate cancer, a prevalent malignancy affecting men globally, has the potential to metastasize to various locations, such as bones, lungs, liver, and lymph nodes [3]. Though metastasis to serous fluids, particularly pleural effusions, is relatively uncommon, it can considerably impact a patient's quality of life and prognosis [3,4]. The rarity of pleural involvement in metastatic prostate cancer has been documented in several case reports and studies [3-5].

Metastatic prostate cancer in serous fluids can pose a diagnostic challenge, as cytomorphology can be nonspecific and may overlap with reactive mesothelial cells or other epithelial and non-epithelial malignancies. Morphologic features of metastatic adenocarcinoma may be variable due to primary tumor histologic type and grade, cellularity, and the presence of reactive or degenerative changes (e.g., therapy-related).

Tumor cells in serous ﬂuid can appear in various forms such as tight clusters, found in breast ductal carcinoma, or as linear arrangements, as seen in breast lobular carcinoma. They might also take the shape of signet ring cells, a characteristic of breast lobular carcinoma and gastric adenocarcinoma, or form papillae, which are common in lung, breast, and thyroid cancers. When it comes to metastatic adenocarcinoma, the predominant origins are the lungs, followed by the digestive system, the pancreatic and biliary regions, and ﬁnally, the genitourinary system [6,7]. In males, the primary locations where malignant pleural effusions frequently occur include the lungs, lymphatic system, digestive organs, and pancreas [6,7].

In the context of prostate adenocarcinoma, the cells typically form small acini, display an individualized arrangement, and possess enlarged nuclei with marked nucleoli [6,7]. However, in this instance, the limited number and singular arrangement of dispersed tumor cells, despite their prominent nucleoli, and the absence of acini structure, provide limited diagnostic insight.

Within the intricate realm of cytopathology, discerning metastatic prostatic adenocarcinoma, particularly in pleural fluid specimens, is often a daunting task. Historically, PSA and PAP/prostatic specific acid phosphatase (PSAP) have served as foundational markers in our diagnostic repertoire. NKX3.1 is prominently expressed in both benign prostate epithelial cells and prostate malignancies, with additional expression noted in testicular tissue and invasive breast ductal or lobular carcinomas [8]. While PSA's sensitivity is recorded at 94.2%, both NKX3.1 and PAP/PSAP align impressively at 98.6%, with NKX3.1 further showcasing a robust specificity of 99.7% [9]. Moreover, the inherent nuclear and diffuse staining attributes of NKX3.1 are particularly invaluable in the cytopathology of pleural fluids [10]. Though PSA and PSAP persist in their essential roles, their synergistic application with NKX3.1 can potentially heighten both sensitivity and specificity. This trio, thus, fortifies our diagnostic toolkit, ensuring we can more confidently and comprehensively pinpoint metastatic prostatic adenocarcinoma in challenging pleural fluid specimens.

Cytologic and immunohistochemical findings provide valuable diagnostic information, allowing for a more accurate and definitive diagnosis of metastatic prostate adenocarcinoma. In this case, morphologic features are nonspecific. Positive immunostaining results for MOC-31, PAP/PSAP, PSA, and NKX3.1 confirmed the diagnosis of metastatic prostate adenocarcinoma.

## Conclusions

Overall, the diagnosis of metastatic prostate cancer in serous fluids may require a high degree of suspicion. Proper diagnostic methods, including cytopathology and immunohistochemical staining, are crucial to ensure timely diagnosis and optimal patient care. Recognition of atypical presentations is essential to avoid misdiagnosis and unnecessary treatment. Despite the poor prognosis associated with malignant pleural effusions, early detection and prompt management can improve the patient's quality of life and potentially prolong survival.

Metastatic prostate cancer with pleural involvement is a rare clinical scenario that can present atypically. Accurate diagnosis relies on cytopathology and immunohistochemical staining, including PAP/PSAP, PSA, and NKX3.1. This case report highlights the importance of considering metastatic prostate cancer in patients presenting with pleural effusions and the need for proper diagnostic methods. A comprehensive literature review of reported cases emphasizes the significance of recognizing atypical presentations of metastatic prostate cancer to ensure timely diagnosis and optimal patient care. Further studies are needed to explore the pathogenesis and clinical implications of this rare occurrence.

## References

[1] Bubendorf L, Schöpfer A, Wagner U (2000). Metastatic patterns of prostate cancer: an autopsy study of 1,589 patients. Hum Pathol.

[2] DiBonito L, Falconieri G, Colautti I, Bonifacio D, Dudine S (1992). The positive pleural effusion. A retrospective study of cytopathologic diagnoses with autopsy confirmation. Acta Cytol.

[3] Knight JC, Ray MA, Benzaquen S (2014). Malignant pleural effusion from prostate adenocarcinoma. Respir Med Case Rep.

[4] Lyonga Ngonge A, Amadife SN, Wireko FW, Ikwu I, Poddar V (2022). A case report on atypical presentation of metastatic prostate cancer. Cureus.

[5] Jeon J, Kim TJ, Park HS, Lee KY (2018). Malignant pleural effusion from metastatic prostate cancer: A case report with unusual cytologic findings. J Pathol Transl Med.

[6] Chandra A, Crothers B, Kurtycz D, Schmitt F (2020). Malignant pleural effusions primary sites and metastatic adenocarcinoma sources in males. The International System for Reporting Serous Fluid Cytopathology.

[7] (2018). Serous effusions - etiology, diagnosis, prognosis and therapy. https://link.springer.com/book/10.1007/978-3-319-76478-8.

[8] Gelmann EP, Bowen C, Bubendorf L (2003). Expression of NKX3.1 in normal and malignant tissues. Prostate.

[9] Gurel B, Ali TZ, Montgomery EA (2010). NKX3.1 as a marker of prostatic origin in metastatic tumors. Am J Surg Pathol.

[10] Wang M, Abi-Raad R, Adeniran AJ, Cai G (2021). Expression of NKX3.1, Prostatic Specific Antigen and Prostatic Specific Alkaline Phosphatase in Cytology Specimens of Metastatic Prostatic Carcinoma. J Clin Transl Pathol.

